# Holographic model of craniosynostosis for HoloLens

**DOI:** 10.1590/acb404825

**Published:** 2025-07-07

**Authors:** Mauricio Mitsuru Yoshida, André Luiz Pires de Freitas, José da Conceição Carvalho, Julio Sergio de Souza, Vinicius Santos Baptista, Lydia Masako Ferreira

**Affiliations:** 1 Centro Universitário da Faculdade de Medicina do ABC – Santo André (SP) – Brazil.; 2 Universidade Federal de São Paulo – Escola Paulista de Medicina – São Paulo (SP) – Brazil.; 3 Intelligenz Robótica – São Paulo (SP) – Brazil.

**Keywords:** Holography, Augmented Reality, Models, Anatomic, Surgery, Computer-Assisted, Craniosynostoses, Craniofacial Abnormalities

## Abstract

**Purpose:**

To develop a holographic skull model of a deformity resulting from craniosynostosis for the HoloLens.

**Methods:**

The methodology for product creation and prototyping was the design thinking structured with the double diamond. A desk survey was conducted with a literature review and an anteriority search. Based on the desk survey results, brainstorming was performed to develop solutions to improve the surgeon’s performance in craniosynostosis using mixed reality.

**Results:**

Reports or scientific articles relating mixed reality use to craniosynostosis were not found in search engines or bibliography databases. A surgeon’s performance potential improvement was observed using mixed reality as an auxiliary tool in craniosynostosis surgery. A craniosynostosis skull hologram was developed in mixed reality, with interactivity commands controlled by gestures, facilitating a three-dimensional spatial understanding of cranial anatomy.

**Conclusion:**

A holographic skull model with a deformity resulting from craniosynostosis was developed for the HoloLens.

## Introduction

Craniosynostosis is defined as the premature fusion of one or more cranial sutures resulting in brain growth restriction and consequent specific patterns of craniofacial skeleton deformity^1^^,^ 2. It is the most frequent cause of cranial deformity in the pediatric age3, with a worldwide incidence of 1:2,000 to 1:2,500 live births^2^^,^ 4 ^,^ 5, and it is the most common cranial malformation that needs surgical treatment in children^6^.

Once it can be related to intracranial hypertension, neurological development delay, hydrocephaly, visual disturbances, language and speaking delay, and Chiari malformation^1^^,^ 2 ^,^ 7-12, the craniosynostosis surgical treatment is recommended to be performed until the first year of age^2^^,^ 13 ^,^ 14.

But its surgery is highly challenging, because there is a relative rarity of the pathology^15^^,^ 16; it demands a deep anatomy familiarity associated with a surgeon’s three-dimensional visualization competence^17^; a complex and altered craniofacial anatomy is presented^15^; and there is a concomitant surgical approach of orbits and cranial bones by intra and extracranial accesses^16^^,^ 18 with two teams (craniofacial surgery and neurosurgery) acting simultaneously^16^^,^ 19. In addition, complications are reported in up to 35.9% of cases^2^^,^ 14 ^,^ 20-27 with mortality rates of up to 2.2% of cases^20^^,^ 22 ^,^ 27-29.

The surgical planning is based on computerized tomographic images^2^^,^ 30-33, which is considered the gold standard exam for craniosynostosis diagnosis^33^^,^ 34. However, the surgeon must mentally relate the two-dimensional tomographic images to the actual three-dimensional anatomy of the patient^17^^,^ 35 ^,^ 36, which requires minimal cognitive spatial visualization ability.

The significant computational development of the last two decades and the need for three-dimensional object visualization in different human activities have driven the development of technologies with the capacity to improve the user’s spatial notion and the ability to superimpose digital information on the real physical world, with emphasis on mixed-reality, which is determined by an environment where multiple users can interact spatial and simultaneously with the virtual objects, offering a perspective and depth experience through a head-mounted display^27^^,^ 37-39.

HoloLens is a mixed-reality device built by Microsoft that works with holograms and combines several types of sensors (infrared lasers, high-definition cameras, accelerometers, and microphones) and an integrated computer^38^. Holographic images, three-dimensional objects, and two-dimensional windows can be positioned at any location in the user’s visual field, allowing for an innovative interactive experience^35^^,^ 38 ^,^ 40-45.

The creation of craniosynostosis holographic models for HoloLens could allow, through the dynamics between the user and the model, better spatial visualization of the patient’s specific anatomy, better identification of changes in the calvaria, better surgical planning, in addition to enabling intraoperative guidance, with real-time access to patient image data in a sterile environment through virtual windows positioned in the operating room^38^^,^ 42.

In this way, using new technologies, such as mixed reality, provides tools to the surgeon to plan the surgeries better and succeed in better operative performance, with consequent benefits to patients.

This study aimed to develop a holographic model for HoloLens of a craniosynostosis cranial deformity.

## Methods

This study is primary, descriptive, and was developed in a single center, with Universidade de São Paulo Research Ethical Committee approval.

A literature review was conducted in the MEDLINE, Latin American and Caribbean Health Sciences Literature (LILACS), Scientific Electronic Library Online (SciELO), and Cochrane databases to obtain relevant scientific articles on the use of mixed reality in surgery or the treatment of craniosynostosis, from January 1, 2015, to May 31, 2021. The keywords and descriptors defined in the database of Health Sciences Descriptors (DeCS) and Medical Subjects Headings (MeSH) were extended reality, mixed reality, augmented reality, virtual reality, holography, smart glasses, HoloLens, surgery, intraoperative, and operative surgical procedures.

The search strategy used was: *((“extended reality”* OR *“augmented reality”* OR *“mixed reality”)* NOT *“virtual reality”)* AND *(holography* OR *“smart glasses”* OR *HoloLens)* AND *(surgery* OR *intraoperative* OR *“operative surgical procedures”)*.

Inclusion criteria were language (English, Spanish, or Portuguese), original articles, systematic reviews with and without meta-analyses, clinical trials, and articles relating mixed or extended reality to surgery. Criteria for non-inclusion were articles published in journal proceedings, letters to the editor, book chapters or monographs, master’s dissertations, or doctoral theses. Articles that related extended reality to academic teaching or patient’s education were not included either. Exclusion criteria were articles that, after being included in the reading, were exclusively related to virtual reality or were duplicated.

The authors would search for solutions to increase surgeons’ performance in correcting craniofacial deformities using mixed reality from the information obtained from the literature research.

## Results

The search strategy identified 100 scientific articles in the MEDLINE database and 10 in the Cochrane database; no articles were found in the LILACS and SciELO databases.

In the MEDLINE database, 43 articles were not included, based on the analysis of the title and abstract, and 57 articles were chosen for reading, of which 34 were selected.

In the Cochrane database, two articles were not included in the title and abstract analysis, and one article was duplicated. Six articles were chosen for reading, of which two articles were selected.

The priority search revealed several experiences using augmented and mixed reality in surgical procedures. At Johns Hopkins University, in the United States of America, mixed reality was utilized for the placement of six pins during spinal fusion surgery and spinal cord resection. At Stanford University, also in the United States of America, and the Champalimaud Foundation, in Portugal, mixed reality was employed for planning breast tumor resection surgery. At Imperial College London, in the United Kingdom, holograms were projected onto the limbs of patients undergoing reconstructive surgery. In the field of orthopedics, a teleconference was conducted between a surgeon in Jaraguá do Sul, Santa Catarina, Brazil, and two remote surgeons in Paris, France, and New Jersey, United States of America. This collaboration focused on the surgical treatment of clavicle fractures, allowing real-time discussions based on the sharing of the surgeon’s field of vision and the visualization of holograms. Additionally, in New Hampshire, United States of America, mixed reality was applied in total hip arthroplasty surgery. No reports of experiences using mixed or augmented reality in surgery for craniosynostosis correction were found.

From the literature research information, the authors decided to increase surgeons’ performance in correcting craniofacial deformities using mixed reality.

A plastic surgeon, a craniofacial surgeon, two neurosurgeons, a graphic designer, and an engineer held brainstorming meetings. After five sessions, holographic models of different types of craniosynostosis were built with adequate response to gestures. Finally, the most representative holographic model of a skull with craniosynostosis was set, with the possibility of interaction with it, based on movement, rotation, and scale functions, to allow the three-dimensional spatial observation of the cranial structure from different angles of view.

**Figure 1:**
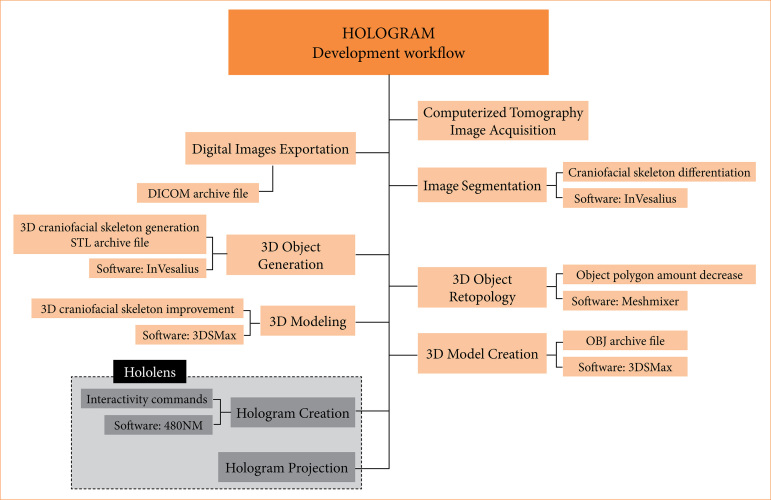
Stages of holographic model development.

Source: Elaborated by the authors.

The development of the holographic model was carried out based on the steps described in Fig. 1.

### Computerized tomography image acquisition

Skull and face images of a patient with craniosynostosis were obtained from a multi-channel medical tomograph with spiral volumetric acquisition without contrast injection and axial slices of 1-mm thickness.

### Digital images export

These digital images were burned to DVD in Digital Imaging and Communications in Medicine (DICOM)–an electronic file format standard for storing medical diagnostic information (X-rays, mammograms, magnetic resonance imaging, and computerized tomography scans)–, and exported to a personal computer (PC, Windows 10, Intel Core^TM^ i7-8565U, 8GB memory, 1TB storage, with NVIDIA GetForce MX110 2GB dedicated card).

### Image segmentation

Image segmentation refers to the process of decomposing a digital image into multiple constituent parts corresponding to objects or topographic regions, aiming to particularize them and facilitate their analysis.

The segmentation was performed using the InVesalius software (Renato Archer Information Technology Center, Campinas, São Paulo, Brazil), which differentiated, through a selection, the craniofacial bone structures from the other structures present in the examination (for example, face mask, orotracheal tube or probes).

InVesalius is a software for reconstructing images from magnetic resonance imaging or computed tomography scans, available for Microsoft Windows and Linux platforms.

### Three-dimensional object generation

In the InVesalius software, a three-dimensional polygonal model of the craniofacial bone structure was created after segmenting the images, containing only the structures of interest, and saved in stereolithography (.STL) file format. Stereolithography is the most widely used standard file for rapid prototyping data transmission that contains data that describes the layout of a three-dimensional object, transforming the surfaces of a solid model into triangles.

This three-dimensional object consisted of a vector model with dimensions in the three axes, representing exclusively the craniofacial bone skeleton, and was exported to the Meshmixer software (Autodesk, San Rafael, CA, United States of America) for retopology.

### Retopology

Retopology involves reducing the number of polygons that make up a three-dimensional object and was performed to increase the model’s interactivity command response.

The software Meshmixer (Autodesk, São Rafael, CA, United States of America) was used for the retopology.

### Three-dimensional modeling

Three-dimensional modeling improves the three-dimensional object, allowing retouching, cutting, subtracting, or constructing object elements.

The three-dimensional modeling was done in the 3DS Max software (Autodesk, São Rafael, CA, United States of America), correcting imperfections, enhancing the three-dimensional object, and choosing its texture.

### Three-dimensional model creation

The cranial models were recorded in an Object File Wavefront (3D.OBJ) file and exported to the 480NM software (Intelligenz Robótica, São Paulo, São Paulo, Brazil), that created the holograms.

Three-dimensional Object File Wavefront is a format used for three-dimensional objects containing three-dimensional coordinates (polygonal lines and points) and texture maps, among other object information.

The models were converted to an .OBJ file, because it is one of the file formats that can be read by the 480NM software installed on the HoloLens.

### Holograms creation

The .OBJ 3D models were exported to the 480NM software installed on HoloLens (Microsoft, Redmond, WA, United States of America), which created the three-dimensional model holograms and their interactivity commands, allowing movement, rotation, and scale control of the objects.

### HoloLens projection

HoloLens, with the 480NM software, projected the holograms and allowed the user to interact with the holographic images through voice commands, gestures, or eye tracking (see the video at https://youtu.be/60hsGnXkuLs).

A 10-month-old male patient with a trigonocephaly craniosynostosis pattern was undergoing surgical planning for the correction of his deformity, during which a holographic model was created.

The creation of the hologram involved acquiring tomographic images with specific requirements, with the thickness of the sections being the most crucial factor in the initial stage of the process.

The 480NM software is not validated for medical purposes; as a result, its use was limited to observing the three-dimensional craniofacial skeleton hologram without the option for reliable measurements. For the same reason, the hologram was not intended to be used as a navigator during the surgical procedure.

During the preoperative planning phase, the surgeons required initial training to become familiar with the device, gestures, and basic functions for interacting with the hologram, which was accomplished without difficulty.

Despite reports of discomfort with the HoloLens due to its weight and ergonomics, using it for short periods was manageable. However, the limited field of vision made it challenging to view objects when they were resized to larger proportions.

In the intraoperative period, technical difficulties arose while dissecting the dura mater in a specific area above the sagittal sinus, in which it was more firmly adhered to the inner surface of the calvaria. The visualization of the patient’s hologram by a surgeon positioned outside the operative field provided a detailed assessment of the craniofacial anatomy, particularly the internal surface of the calvaria. This visualization helped identify bony protrusions and prominences near the relevant suture, which led to a modification of the surgical strategy for the safe dissection of the dura mater.

## Discussion

Compensatory deformities resulting from craniosynostosis can change the craniofacial skeleton in different ways and at varying levels of severity, and occasionally, more than one suture may be involved, resulting in distinct three-dimensional craniofacial configurations.

Due to these changes in the complex craniofacial anatomy, the surgeon’s accurate three-dimensional vision is fundamental for adequate surgical planning, precise execution of the procedure, and its success.

Many craniofacial surgical procedures are based on complementary imaging exams, such as computerized tomography and magnetic resonance imaging, and are analyzed on two-dimensional screens. Even the so-called three-dimensional reconstructions of these exams do not present themselves in three dimensions in space since they are visualized on two-dimensional computer screens.

Therefore, the three-dimensional mental visualization of the craniofacial skeleton requires a cognitive interpretation and construction of the real anatomy from two-dimensional image data. In addition, during surgery, it is often necessary to consult complementary imaging exams outside the operative field to calibrate the actual anatomy found with the surgeon’s mental image, which disperses his attention from the surgical site and contributes to fatigue and increased operative time, in addition to increasing the potential risk of error related to task switching^35^.

In addition, such images are not arranged according to the surgeon’s perspective of vision; therefore, imagination and experience are required to understand and mentally project the images onto the patient’s body during the surgical procedure^46^. Moreover, there is a limitation to ensuring sufficient accuracy of the surgeon about the spatial position of the anatomical structures^47^, since there may be a gap in the transfer between the images of the exam, the surgeon’s mental image, and the actual anatomy of the patient^48^, with relevant information losses, from the planning process until the execution of the surgical procedure^49^, increasing the morbidity of the surgery.

Technological advances have markedly impacted the treatment of craniosynostosis, with improvements in the processes of diagnosis, anthropometric measurement, preoperative planning, intraoperative procedure, interpersonal communication, and medical education^3^. Currently, computer-assisted surgery technology applied to craniosynostosis allows the creation of stereolithographic models, surgical simulation of osteotomy patterns and bone repositioning, and the creation of cutting guides and customized implants^50^.

Printed three-dimensional anatomical prototypes are considered the gold standard models for spatial visualization and surgical planning^51^. However, they have a high-production cost and relatively long printing time.

Nevertheless, new technologies are still being developed, and extended reality stands out. Extended reality is a generic term that refers to all environments in which real and virtual elements are associated to simulate or improve the physical world, including virtual, augmented, and mixed reality^52^.

Virtual reality is the technology that allows the creation of a totally artificial and physically non-existent world by the computer, in which the user is immersed in a wholly digital environment not connected to the real world^52^. Augmented reality combines virtual objects and the real world by superimposing these digital elements on the actual environment to increase the user’s perception of the world. Superficial interactions between the user and virtual objects are possible, but limited^52^, since interactivity occurs through two-dimensional screens of cell phones or tablets. Finally, mixed reality is considered the fusion of the best features of virtual reality and augmented reality and corresponds to the fusion of the real and digital worlds, with the coexistence, in space and real time, of virtual objects (holograms) and concrete objects. With this technology, the user can interact and manipulate virtual objects since they are spatially responsive^52^.

HoloLens is a mixed-reality device created by Microsoft in 2015 that projects holograms and allows multiple users to interact simultaneously with virtual objects. It is equipped with infrared light sensors, high-definition cameras, an accelerometer, and a microphone, with a wireless system, Windows Holographic, 4GB RAM and 64GB memory, eye tracking, voice, and gesture commands, with the image quality of 2K per eye, field of view of 43 degrees on the horizontal axis and 29 degrees on the vertical axis.

HoloLens was the first commercially available mixed-reality device approved by the U.S. Food and Drug Administration in 2018 for preoperative planning.

The present research describes a workflow for holographic model creation in mixed reality, patient-specific, generated from computerized tomography data to assist the surgeon in planning and executing surgeries for craniosynostosis correction. The hologram-creating process presented in this work uses computer-aided design (CAD) software, a technology initially applied in the automotive and aeronautical sectors and later expanded to the areas of engineering and architecture and is currently being increasingly used in medicine due to the development of three-dimensional technologies.

There are indications that holographic representation is better than representation on two-dimensional screens for the spatial understanding of anatomy since visualization in three dimensions, under different viewing angles, increases the perception of form and depth, decreases the mental load, and allows completing tasks faster and with better quality of results^51^. In addition, there is an increase in three-dimensional understanding once mixed reality still allows the holographic model to be rotated and moved.

In craniosynostosis preoperative surgical planning, the use of mixed reality could improve the patient’s anatomy visualization with its specific variations^53^^,^ 54, potentially reducing the difficulty in anatomical three-dimensional understanding^55^ and decreasing surgical planning time^48^. The simultaneous interaction of multiple users with the same holographic model^42^^,^ 56 would contribute to the interdisciplinary discussion and definition of the best surgical strategy.

Notably, during the surgical procedure, mixed reality would allow the surgeon to place virtual objects anywhere in his field of vision, with complementary information visualization and patient data consultation, without leaving the operative field, maintaining the interaction with the surgical site and preserving the attention and focus on the patient^46^^,^ 57 ^,^ 58. This could reduce surgical procedure morbidity since there is evidence that the rupture of the visual-motor axis during surgery can cause ergonomic problems, decrease surgical performance, cause spatial disorientation, and increase the risk of iatrogenic intraoperative injuries^47^. Quick access to complementary information could also help intraoperative decision-making, reducing the surgeon’s stress^59^. In addition, a hologram of the complete virtual surgical planning (with osteotomy lines, ideal positioning of bone segments, and final cranial shape) could be projected onto the patient, serving as an intraoperative guide, in addition to providing intraoperative navigation to improve spatial orientation and assist in noble anatomical structures localization^60^ in a patient-specific condition.

Once the holographic models are virtual objects inserted in the surgical field, they would have the advantage of keeping the process completely aseptic, allowing the surgeon to keep his hands free for the surgical instruments^58^. Moreover, the use of mixed reality in surgery could improve the accuracy, safety, and efficiency of surgical procedures^45^ and, from an economic point of view, reduce operating room occupancy time and hospital costs^42^.

Although there have been no reported applications of mixed reality in craniosynostosis, this technology has already been utilized in various medical fields, including cardiac interventions^61^, orthopedic surgeries^62^, and mandibular osteotomies in oncological procedures^47^. However, many of these studies remain in experimental or proof-of-concept phases and have primarily been validated using prototypical models, such as phantoms or cadavers^63^. Additionally, these studies often lack robust designs and consistent regulatory standards, making it difficult to draw statistically significant conclusions when compared to other technologies^64^^,^ 65.

Although mixed reality has incredible potential for professional practice, particularly in surgical planning and surgery execution, the technology still has limitations.

The current HoloLens hardware is bulky and heavy, which limits its use by surgeons during lengthy intraoperative procedures, potentially causing discomfort such as nausea, disorientation, headaches, eye strain, and dry eyes.

A learning curve for the efficient use of voice and gesture commands is required^42^, and voice commands have specifically more limited control for operating room use due to the characteristic sound environment of the space^45^, with noise that can cause interference. The experience and familiarity of surgeons with digital technologies can impact their learning curve. Older surgeons, in particular, may find it more challenging to integrate mixed-reality tools into their daily practice. Additionally, incorporating mixed-reality technology into the operating room adds complexity, which can create a steep learning curve for surgical teams. This challenge may affect the efficiency and safety of surgical procedures during the initial implementation, underscoring the necessity for proper training programs for users.

The battery life of up to 3 hours of active use can be also a limiting factor for more complex surgeries^46^. In addition, the simultaneous interaction of multiple users with the same holographic model requires a robust wireless network for data connection, which can be a restrictive condition in several locations.

While HoloLens and similar devices offer promising new dimensions for surgical planning and intraoperative assistance, a comprehensive regulatory compliance strategy must address the certification under relevant safety standards and regulatory clearance if used as a medical device, adherence to strict cybersecurity and data protection regulations, rigorous software validation and clinical workflow integration, extensive user training and documented risk management procedures, and environmental and operational factors in the clinical setting.

Despite the challenges limiting the widespread adoption of mixed reality in surgical procedures, ongoing research and the development of technologies will address these obstacles.

## Conclusion

A holographic skull model with a deformity resulting from craniosynostosis was developed for the HoloLens providing the observation of an image as a three-dimensional object in the user’s actual environment, allowing the visualization of all the peculiarities of the craniofacial anatomy from all viewing angles, including inside views, using the utility functions “move”, “rotate”, and “scale.”

## Data Availability

Data sharing is not applicable.
